# First Report of Colleters in Araceae: A Case Study in *Anthurium andraeanum* Reveals Diverse Mucilage Glands Associated with the Developing Shoot

**DOI:** 10.3390/plants12162912

**Published:** 2023-08-10

**Authors:** Carlos Gabriel Pereira-Silva, Igor Ballego-Campos, Cássia Mônica Sakuragui, Eduardo Gomes Gonçalves, Elder Antônio Sousa Paiva

**Affiliations:** 1 Departamento de Botânica, Instituto de Ciências Biológicas, Universidade Federal de Minas Gerais, Belo Horizonte 31270-901, Brazil; c.gps2219@gmail.com (C.G.P.-S.); igorballego@gmail.com (I.B.-C.); 2 Departamento de Botânica, Instituto de Biologia, Centro de Ciências da Saúde, Universidade Federal do Rio de Janeiro, Rio de Janeiro 21941-902, Brazil; cmsakura12@gmail.com; 3 Yamandu Biofilia e Paisagismo, Itacaré 45530-000, Brazil; edggon@gmail.com

**Keywords:** mucilage, plant–environment interaction, aroids, desiccation, programmed cell death

## Abstract

Araceae comprises a diverse group of plants that grow in various habitats, ranging from submerged aquatics to lithophytes. Thus, aroids are likely to show diverse glands acting in several plant–environment interactions, including colleters that protect young shoots. Based on this premise and the lack of studies regarding secretory structures in Araceae, we employed standard light and electron microscopy methods to test the hypothesis that colleters are present in *Anthurium*. Our main goals were to identify mucilage glands in *A. andraeanum* by conducting a detailed anatomical study of their structure, ultrastructure, and secretory activity. We found finger-like colleters in the apex of young leaves, spathes, and unexpanded cataphylls as well as secreting zones at the apex of expanded cataphylls, at the margins of non-fused cataphylls, and throughout the keels in two-keeled cataphylls. The colleters develop precociously and senesce shortly afterwards. Ultrastructural data and histochemistry confirmed the production of a polysaccharide-rich secretion that fills the spaces within the developing shoot. As far we know, this is the first time that colleters have been reported for Araceae. The functional roles of the secretion and the position of finger-like colleters concerning the ‘precursor tip’ of monocotyledons are discussed. Future research correlating secretory activity in colleters of species from different habitats might reveal a great diversity of mucilage glands with ecological and evolutionary significance to the family.

## 1. Introduction

One of the most diverse families of monocots, the Araceae, comprises about 3700 species [1,2] that grow in a wide array of habitats, ranging from submerged aquatic plants to lithophytes adapted to exposed rock outcrops. As a result of this great diversity and adaptive capacity, Araceae species would be expected to show a diverse range of secretory structures involved in several distinct plant–environment interactions. Evidence for secretion-mediated interactions in aroids includes the observation of various exudates, such as nectar, stigmatic secretions, and volatile fragrant substances (see [3] and references therein). However, the source and chemical nature of these exudates are often only indirectly inferred, and the anatomy and secretory activity of the secreting structures still need to be explored. This practice renders the knowledge of secretory systems in Araceae deficient and imprecise, making it difficult to understand interactions mediated by aroid glands or the evolutionary relationship between these structures [3]. Despite the diversity of glands within the family, records of their occurrence are restricted to a few species and often lack detailed studies. Among the secretory structures reported for Araceae, there are records of hydathodes [4], extrafloral nectaries [5,6], floral nectaries [3], laticifers [7], mucilage cavities [7], osmophores [8,9], and resin canals [7].

Not surprisingly, secretory structures remain largely understudied within *Anthurium*, the largest aroid genus [2] with ca. 1500 estimated species. This megagenus is also morphologically and ecologically diverse, spreading through distinct areas of the neotropics [10]. However, even aspects related to its floral biology are still insufficiently known, and the controversial presence of floral nectaries is an example of the traditional problems regarding secretory systems in this genus [3].

Colleters are plant secretory structures associated with young organs, exuding a mucilaginous, mixed, or resinous secretion [11,12]. Many of the functions attributed to colleters are controversial and may be restricted to particular cases; lubrication and protection of meristems and young organs, protection against desiccation, the facilitation of symbiotic processes, and protection against herbivores stand out as the most studied (see [13] and references therein). Among these functions, protection against excessive water loss seems to be the most relevant, as suggested by several authors and in different taxonomic groups [14,15,16,17]. Colleters present great structural diversity, ranging from simple trichomes to complex structures [14].

The record of the presence of colleters in monocots is relatively recent [18] and is restricted to Bromeliaceae [19], Orchidaceae [18,20,21,22,23], and Rapateaceae [24]. According to Cardoso Gustavson et al. [21], the structure of colleters in monocots has rarely been studied, making it difficult to interpret their ecological roles and evolutionary history. Colleters in monocots are scarce [20], but there are reports of “axillary squamules” that supposedly secrete protective mucilage (see [25] and references therein). These squamules and their relationship with mucilaginous secretions are reported for Araceae (see [8] and references therein). Considering the diversity of taxa in Araceae and their wide occurrence worldwide, we believe that the poor understanding of colleters in the family is a consequence of the lack of adequate anatomical and structural studies. Many *Anthurium* species are epiphytes and, therefore, live in conditions of unstable water supply, which, in addition to the large size of their leaves, makes them susceptible to water loss. Thus, some protection against excessive transpiration is expected, especially in young portions of the plant. The secretion produced by colleters is considered to be effective in protecting immature organs, especially leaves, against excessive water loss [14,17].

Based on these premises, we hypothesize that colleters are to be expected in *Anthurium* species. To test this hypothesis, we searched for evidence of protective secretion in *Anthurium*, looking for colleters in a detailed anatomical study with *Anthurium andraeanum*. Our main goals were (a) to identify potential colleter-like glands occurring in the vegetative and reproductive axis of *A. andraeanum* and (b) to conduct a detailed anatomical study on the structure, ultrastructure, and secretory activity of these glands. 

## 2. Results

### 2.1. Structure and Histochemistry

**Figure 1:**
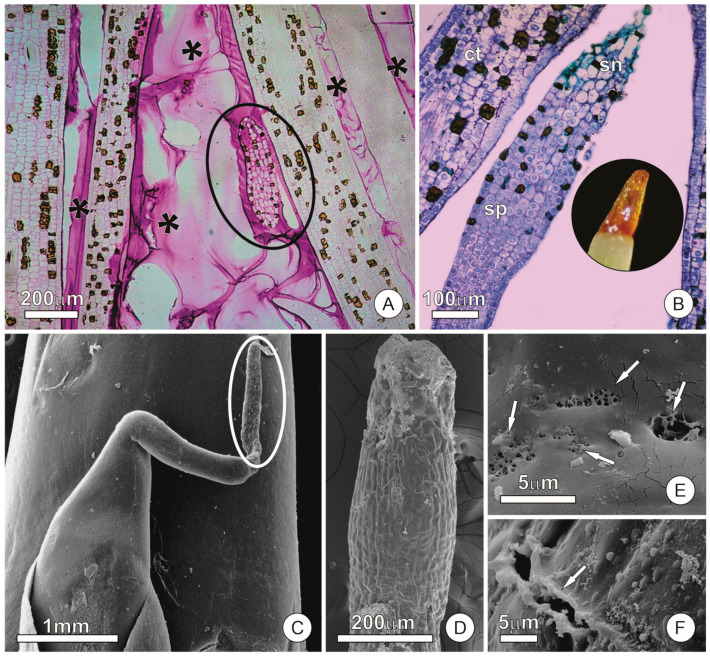
Light and scanning electron micrographs showing the finger-like extension in the apex of the leaves and spathes of *Anthurium andraeanum* and details of its cuticle. Sections stained with periodic acid–Schiff (PAS) reagent and toluidine blue. (**A**) Longitudinal section of a leaf apex showing the finger-like colleter (highlighted) and another foliage leaf immersed in mucilage (asterisks). (**B**) Longitudinal section of a leaf internal to a cataphyll showing the colleter at the apex. Note that the distal portion of the colleter in the image is already in senescence, whereas the basal portion is still secretory. The cells in the secretory portion show a dense cytoplasm and voluminous nuclei, while the cells in the senescence portion show an increase in the vacuome and a loss of shape. In detail, a stereomicroscope image of a leaf apex showing the distal portion of the finger-like colleter in senescence (brown color) (**C**) Colleter (highlighted) at the apex of a spathe. (**D**) Detail of a colleter showing the senescence on the distal portion, evidenced by the loss of turgor and wrinkling of the apex. (**E**,**F**) Details of pores and breaks (arrows) observed in the cuticle. Abbreviations: ct, cataphyll; sn, senescence portion; sp, secretory portion.

The leaves of *A. andraeanum* present an entire leaf blade with an acuminate apex. At the shoot apex, foliaceous organs include cataphylls, foliage leaves, and spathes. The cataphylls vary greatly in shape, size, and position; they may be keeled, non-keeled, with margins fused or not fused, or significantly reduced and undifferentiated within accessory buds. The overlapping of these foliaceous organs creates a sequence of closed spaces where the shoot gradually expands. These spaces are filled with a mucilaginous secretion that appears hyaline, quite fluid, and sticky (Figure 1A). The tests with ruthenium red showed negative results for this secretion. In contrast, the periodic acid–Schiff (PAS) test was positive, evidencing a mucilaginous exudate comprising mainly neutral polysaccharides (Figure 1A). The test with Sudan Red B showed negative results for lipids. 

At the apex of each foliage leaf there is a finger-like extension that is about 2–3 mm long (Figure 1A–C). The uppermost portion of this extension (ca. 1 mm) is formed by numerous cells with a dense cytoplasm and voluminous nuclei constituting a colleter (Figure 1A,B). These same colleters also occur at the apex of spathes and unexpanded cataphylls within accessory buds, being similar in size and structure to those on the foliage leaves. The finger-like colleters are cylindrical or slightly conical in shape, subsessile, with a hardly distinct peduncle (Figure 1B,C) that makes these structures quite inconspicuous and easily mistaken by a mere extension of the apex of the organ in which they occur. The absence of vascularization is a striking feature of these structures; the vascular tissues are extinguished shortly before the base of the colleter. 

The colleters differentiate precociously, and regardless of the organ to which they are associated, they are found to be secretory only in very young stages (about 5 mm long foliage leaves, cataphylls, and prophylls). They are hyaline and hardly visible, turning dark shortly thereafter and assuming a brown color. Darkening of the colleters results from evident cellular signs of senescence, which begins in the distal portion of the colleter and progresses towards the base. The first sign of senescence is an increase in the vacuome (Figure 1B), which later progresses to cell death and collapse, resulting in loss of turgor and a consequent change in shape, making the colleter wrinkled (Figure 1D).

The epidermis of the colleter is one-layered and consists of almost cubic cells. The surface of the functional colleter is slightly wavy, stomata-free, and covered by a thin cuticle (Figure 1B). The cuticle was relatively intact in the analyzed samples, with rare pores and breaks (Figure 1E,F).

**Figure 2:**
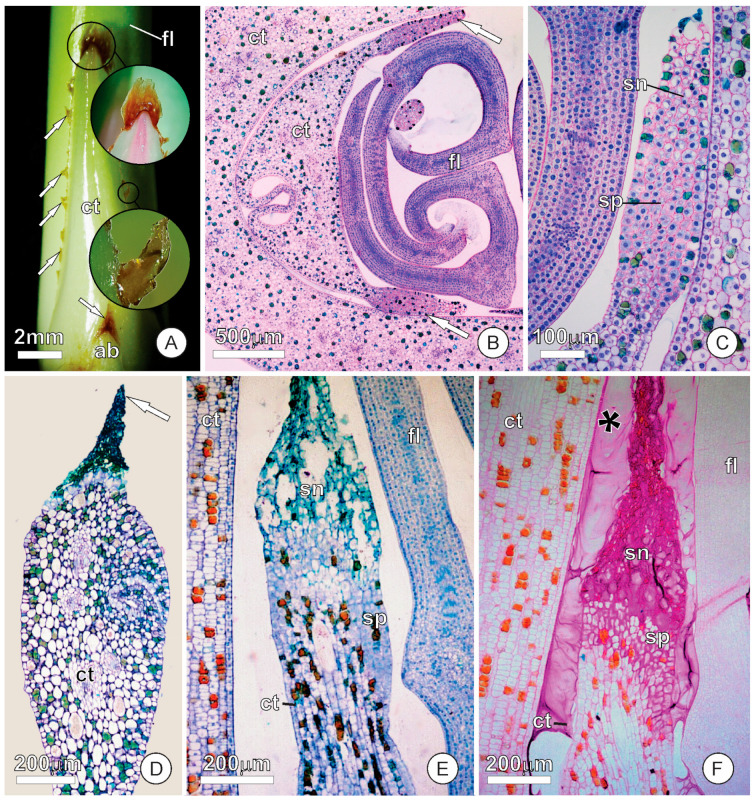
Stereomicroscope image from the cataphyll and light micrographs showing the colleter-like secreting zones in the apex of expanded cataphylls and margin of non-fused cataphylls of *Anthurium andraeanum*. Sections stained with toluidine blue and periodic acid–-Sschiff (PAS) reagent. (**A**) Two-keeled cataphyll with the colleter-like zones in the keels (arrows). (**B**) Transversal section of a two-keeled cataphyll with coleter-like zones (arrows) at the margins. (**C**) Detail of a secreting zone from a cataphyll with the beginning of senescence in the distal portion. (**D**) Cataphyll with a secreting zone after senescence (arrow). (**E**) Detail of a secreting zone from a cataphyll with the beginning of senescence in the distal portion, whereas the basal portion remains secretory. (**F**) Cataphyll with a secretion zone in the apex stained with periodic acid–Schiff (PAS) reagent. Note the secretion (asterisk) around the cataphyll. Abbreviations: ab, accessory bud; ct, cataphyll; fl, foliage leaf; sn, senescence portion; sp, secretory portion.

Aside from the apical finger-like colleters, the cataphylls of *A. andraeanum* can develop distinct colleters during their expansion and differentiation (Figure 2A). These colleters develop as secretory zones at the apex of expanded cataphylls, at the margins of non-fused cataphylls, and throughout the keels in two-keeled cataphylls (Figure 2A,B). They also comprise dense cells with voluminous nuclei that degenerate, as observed in the finger-like colleters of leaves, spathes, and unexpanded cataphylls (Figure 2C–F). The shape of this secreting zone is similar to that of the finger-like colleters (Figure 2B,C). Still, it is not cylindrical, and it actually extends throughout the margins or keels as a membranaceous band that appears as scale-like appendages upon senescence (Figure 2A). The tests with the PAS reagent showed that this secreting zone is also polysaccharide-rich and might contribute to the mucilage that fills the spaces within the shoot apex (Figure 2F).

### 2.2. Ultrastructure and Mucilage Release

**Figure 3:**
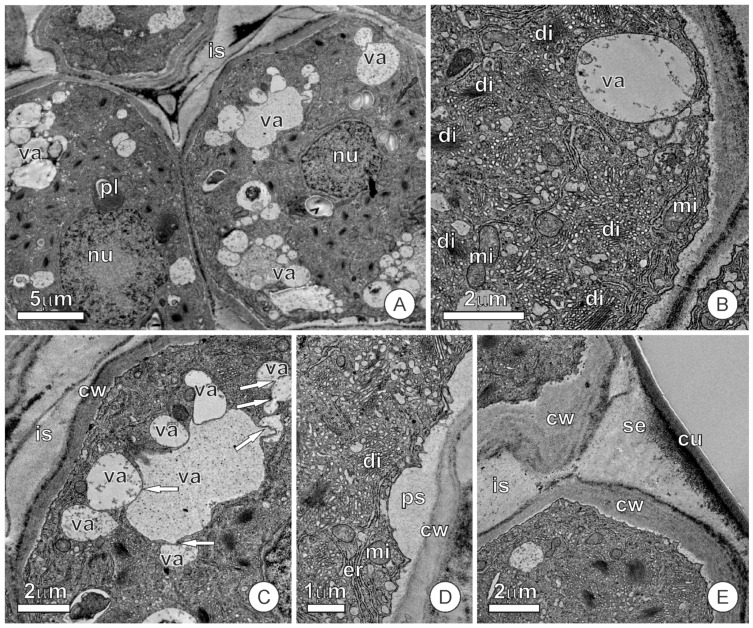
Transmission electron micrographs of the mucilage-secreting cells of the colleter during the secretory phase. (**A**,**B**) Cells show a dense protoplast with an organelle-rich cytoplasm. Note the large number of dictyosomes (in (**B**)) producing vesicles that fuse to form small vacuoles. (**C**) Vacuoles containing mucilage merge with each other (arrows). (**D**) Vesicles and small vacuoles fuse with the plasma membrane, releasing the secretion in the periplasmic space. (**E**) Secretion accumulated in the intercellular space. Abbreviations: cu, cuticle; cw, cell wall; di, dictyosome; er, endoplasmic reticulum; is, intercellular space; mi, mitochondria; nu, nucleus; pl, plastid; ps, periplasmic space; se, secretion; va, vacuole.

Except for the position and presence of the cuticle, the epidermal and internal cells are structurally similar (Figure 3). Thus, we will describe the secretory cells as a whole without reference to their position. 

The cells of the colleter at the secretory stage show a dense protoplast with conspicuous nuclei and an organelle-rich cytoplasm, in which mitochondria, dictyosomes, a rough endoplasmic reticulum, and small vacuoles prevail (Figure 3A–C). The vacuoles seem derived from the fusion of numerous vesicles originating from dictyosomes. We also observed fusion between vacuoles (Figure 3C) and evidence of vesicles and vacuoles fusing with the plasma membrane and releasing their content into the periplasmic space (Figure 3D).

During the phase of intense secretory activity, exudate accumulation can be observed inside the vesicles and vacuoles in the periplasmic space (Figure 3C,D) and the intercellular spaces. However, during this phase, the formation of subcuticular spaces seems restricted, occurring mainly in the direction of the cell junction and creating small spaces (Figure 3E) only observable with transmission electron microscopy (TEM).

In the secretory phase, the dictyosomes present an intense formation of vesicles dispersed throughout the cytoplasm, giving it a granular appearance (Figure 3B,D). Still, at this stage, the smooth endoplasmic reticulum is well-developed, especially in the peripheral portions of the protoplast.

**Figure 4:**
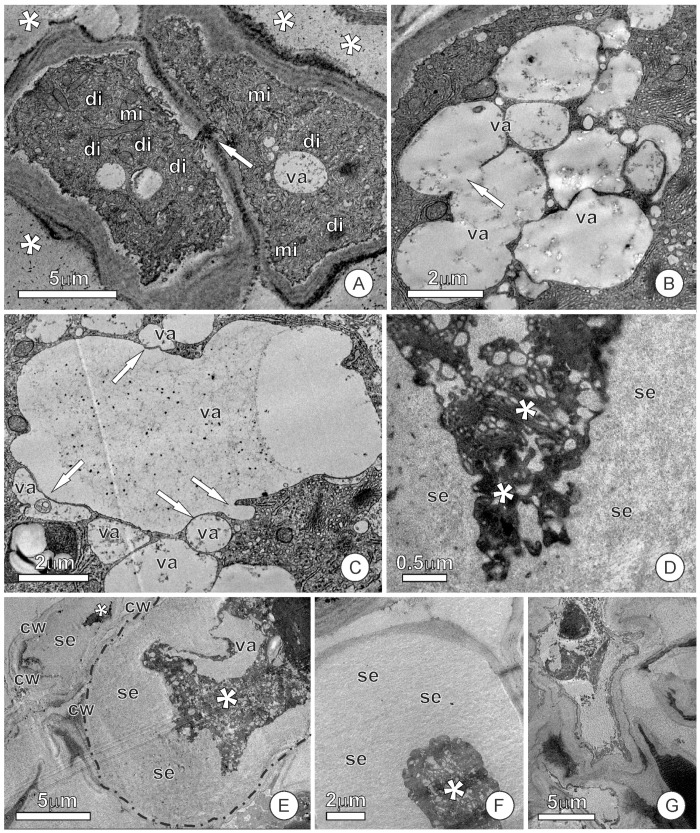
Transmission electron micrographs of the mucilage-secreting cells of the colleter after the beginning of the senescence. (**A**) Image showing a significant amount of exudate accumulated in the intercellular space (*). Note the plasmodesmata (arrow) on the cell wall connecting the cells. (**B**,**C**) The fusion of vacuoles (arrows) generates larger vacuoles with a similar content observed in the intercellular space. (**D**–**F**) Cells with the periplasmic space enlarged by the release of the content that was inside the vacuoles. Note that the protoplasts (asterisks) are very compressed and reduced, while the secretion occupies ample space inside the cell. (**G**) Cell with a considerable loss of shape. Abbreviations: cw, cell wall; di, dictyosome; mi, mitochondria; se, secretion; va, vacuole.

In the cells close to the apex, in which senescence begins, an increase in the volume of intercellular spaces can also be observed with TEM with a significant accumulation of exudates (Figure 4A). In this phase, the fusion of vacuoles appears to be more substantial, generating larger vacuoles in which the content is structurally similar to that observed in the intercellular spaces (Figure 4B,C). In cells that show the first signs of senescence, the release of vacuolar and vesicular contents enlarges the periplasmic space and makes the protoplast compressed, dense and dark, and placed in the center of the cell (Figure 4D–F). The plasma membrane resists at this stage of compression and darkening of the protoplast, and many organelles, such as mitochondria, plastids, and the endoplasmic reticulum, can still be recognized. As this process of synthesis and the accumulation of exudates proceeds, the cell walls become loose and virtually indistinguishable from the large mass of material accumulated in the periplasmic and intercellular spaces (Figure 4E–G). 

This stage of significant protoplast compression seems irreversible, resulting in the vesiculation of organelles that gradually lose their shape and become indistinct. At the end of the process, protoplast residues form clusters of shapeless and black masses amidst the exuded material and remnants of cell walls; there is no evidence, in the images analyzed at this stage, of the significant release of exudates to the outside of the colleters.

## 3. Discussion

Secretory structures in leaf apexes or the tips of marginal teeth are not unusual, with records including extrafloral nectaries, hydathodes, and colleters [26,27,28,29]. Specifically, the presence of colleters in leaf apexes or marginal teeth is reported for Aquifoliaceae, Caryocaraceae, Celastraceae, Euphorbiaceae, Lechytidaceae, Ochnaceae, and Turneraceae [16,29,30,31], thus comprising a common position for foliar colleters. In Araceae, there is evidence of hydathodes at the leaf tip of *Colocasia esculenta* L. [4,27]. 

In *Anthurium polyschistum*, *A. clavigerum*, and *A. pentaphyllum*, Henriquez [32] observed the occurrence of a precursor tip at the apex of the leaves, which is a reduced and unifacial upper leaf zone found on many monocotyledons (but not exclusively). These precursor tips are also known as *Vorläuferspitze*, which is usually a rudimentary cylindrical extension at the apex of the leaf formed via the segmentation of the leaf primordium during the morphogenesis of some leaves [32,33]. Interestingly, our results showed that the colleters coincide in position with the precursor tip in the foliaceous organs of *A. andraeanum*; however, it is important to note that the colleter is not a *Vorläuferspitze*, but rather an apical portion of these precursor tips, extending from a third to half its total length. According to Henriquez [32], the *Vorläuferspitze* of *Anthurium* spp. appears in the early stages of leaf differentiation and shows early signs of necrosis at its apex, reinforcing the similarity with the colleters studied here.

Although the presence of a *Vorläuferspitze* seems to constitute a key trait of some monocotyledonous groups, its function remains unclear. Regarding the possible roles of this structure, Kaplan [33] states: “In addition, Gentner showed that, in different species, the “*Vorläuferspitze*” may have several different functions during leaf development, including mucilage secretion, soil penetration, and even control of expansion of the lamina”. In Araceae, this structure is not widespread, their being absence reported for the *Amorphophallus* [32] genus, in which colleters are absent, as observed in *A. bulbifer* (Paiva EAS, personal observation). Even in cases where this distal portion of the leaf primordium is recognizable as a *Vorläuferspitze*, the presence of a colleter is not always observed.

To our knowledge, this is the first time a colleter has been identified as part of the precursor tip of Araceae. For the first time, a secretory tissue was identified in association with the margins and kells of cataphylls in Araceae. Scale-like appendages similar to those observed in mature cataphylls also occur in *Philodendron*, *Cryptocoryne*, and *Lagenandra* (see [8] and references therein); they might constitute evidence of the same secretory system in these taxa.

The processes of protoplast darkening and evidence of programmed cell death that we observed occurring in *Anthurium* colleters appear to be recurrent processes in colleters that develop in long-lasting organs such as leaves. In the case of colleters arranged in ephemeral structures, such as some stipules, senescence of the colleters does not always occur since these structures suffer abscission, naturally interrupting the metabolic activity in everything connected [31]. Although Henriquez [32] referred to necrosis in the precursor tip of *Anthurium* species, we found evidence suggesting that the senescence of the colleters occurs by programmed cell death (PCD). During the compression and darkening of the protoplast, the integrity of vesicles, mitochondria, and the maintenance of plasma membrane integrity we observed was consistent with the occurrence of PCD, as reported by some authors [34,35]. Programmed cell death is a usual process in the development of plant organs [35,36]. Evidence of the occurrence of programmed cell death in colleters was reported by Mangalan et al. [37] in *Gardenia gummifera* (Rubiaceae) and by Gonçalves et al. [13] in *Prepusa montana* (Gentianaceae). According to Paiva [31], there is evidence that dictyosomes produce lytic enzymes, assuming lysosomal activity and contributing to cell death in colleters. Despite these observations, it is important to note that the knowledge of PCD processes in plants is relatively poor, and morphological evidence might not be sufficient to discuss the events of cell death properly. As with animals, different modes of PCD seem to occur, although confident recognition, identification, and unified terminology are still in debate [38,39]. Thus, further research, including molecular studies and current functional genomics methods, might corroborate PCD and reveal specific modes through which cell death might be regulated in the secretory structures of *A. andraeanum.*

The pores and ruptures that we observed occurring in the cuticle of the colleters, because they are scarce and small, do not seem to give vent to all the exudate produced by these glands. Thus, during the initial phase of the secretory process, the most fluid portion of the mucilage could be released through the cuticular pores; however, a significant part of the exudate remains in the intercellular and periplasmic spaces. The accumulation of exudate, especially in the periplasmic space, seems to trigger the PCD process, culminating in the expressive accumulation of secretion in the lumen of cells, as observed in mucilaginous idioblasts (see [40]). Thus, the apical portion of the colleters, even after the secretory process has ended and it has become brown and withered, seems to contribute to the release of mucilage into the spaces in which the young organs develop. The mucilage, being highly hydrophilic and expandable, after cell death and in the humid atmosphere of the spaces delimited by the cataphylls expands and disperses to the external environment; in fact, after the emergence of these organs, the colleter is nothing more than a dry filament with essentially empty cells. The necessary degradation of the colleters for the exudation of mucilage also explains why the senescence process begins at the very early stages of development. 

The histochemical test using the periodic acid–Schiff reaction revealed a large amount of secretion around the colleters. Also, it allowed the identification of the chemical nature of the secretion, which is mucilaginous and composed mainly of neutral polysaccharides. Considering that the mucilage in eudicots is predominantly formed by acidic polysaccharides [41], the discussion about the presence of neutral polysaccharides in some monocots seems relevant and deserves attention. In Orchidaceae, in some rare reports of colleters in monocots, neutral polysaccharides in the exudates of these glands are reported for *Rodriguezia venusta* (Lindl.) Rchb.f. [18] and *Oncidium flexuosum* Sims. [20], although acidic polysaccharides have been described in the exudates of colleters from *Elleanthus brasiliensis*, another Orchidaceae [15]. In fact, the primary cell wall of some monocots, mainly some representatives of Poales, have low galacturonan contents [40]; however, this is not a rule for monocots, and even in Araceae there are reports of high uronic acid contents, as in *Alocasia macrorrhiza* (L.) Schott [42]. Still, it is necessary to consider that monocotyledons present a reduced pectin content in their cell walls [43], and the nature of the secretion of colleters in this group may be related to the composition of non-cellulosic carbohydrates in the cell wall, whose acidic polysaccharide content is notably variable.

A mucilaginous secretion covering young portions of the axis is often associated with protection against desiccation [14,15,28,44]. In these cases, the hygroscopic nature of the exudate maintains a humid environment, avoiding excessive transpiration and desiccation of the tissues, especially during exposition to the environment. In young organs, the effects of water stress might be more dramatic due to inadequate water supply via the xylem and the absence of other protective structures, such as a thick cuticle layer [14]. In addition, the secretion observed in *A. andraeanum* might be associated with the lubrication of the numerous foliaceous organs during the expansion of the shoot. A similar role for a mucilaginous secretion has been suggested for other congested developing axes [18,19].

## 4. Material and Methods

### 4.1. Plant Material

Five mature specimens of *Anthurium andraeanum* were obtained from two distinct populations growing on the Campus of the Universidade Federal de Minas Gerais (Belo Horizonte, Brazil) after the beginning of the rainy season (October–March) in the years of 2021 and 2022. The plants grew in bright indirect sunlight at two close sites exposed to rain and temperature fluctuations. During the study period, the amount of precipitation was 1638 mm and the mean monthly temperatures ranged from 21.9 °C to 24.3 °C (meteorological station A521, 1.6 km from the study sites; mapas.inmet.gov.br). From each specimen, we sampled at least three portions of the shoot apex, young and mature leaves, and young inflorescences.

### 4.2. Prospection

Young and mature leaves and dissected portions of the shoot apex obtained from the sampled individuals were carefully observed in the field or under a stereomicroscope. We searched for potential secretory structures (e.g., trichomes, emergences, and gland-like structures) in the apex of the leaves, cataphylls, and young spathes and for the presence of viscous fluids that could indicate secretion covering these parts. The presence of potential secretory structures was recorded, and images were documented using a stereomicroscope (Leica, Stemi 2000-C) coupled with a digital camera (Canon, Powershot A650 IS).

### 4.3. Structural Analysis

#### 4.3.1. Light Microscopy

Based on the preliminary prospection, we sampled five stem apices from different individuals of *A. andraeanum*. Fragments of the stem apices were obtained in longitudinal and transversal slices (about 2 mm wide) and fixed under a slight vacuum in Karnovsky’s solution (pH 7.2 in 0.1 M phosphate buffer; modified from Karnovsky [45]) for 24 h. The fixed fragments were then dehydrated in an increasing ethanol series and embedded in synthetic resin (2-hydroxyethyl methacrylate, Leica^®^, Heidelberg, Germany). Thin sections (5–8 mm thick) were obtained on a rotary microtome (Hyrax M40, Carl Zeiss Mikroskopie, Jena, Germany), stained with toluidine blue (pH 4.7 in acetate buffer, modified from [46]), and mounted in glass slides using Entellan^®^ (Sigma-Aldrich, St. Louis, MO, USA). The prepared sections were analyzed using a CX41RF microscope (Olympus Scientific Solutions, Waltham, MA, USA) coupled with a digital camera/image capturing system (TV0.5XC-3, Olympus Scientific Solutions, Waltham, MA, USA). 

Additionally, fresh samples and unstained microtome sections were submitted to histochemical tests with periodic acid–Schiff (PAS) reagent for neutral polysaccharides [47], ruthenium red (0.002% aqueous solution) for acid polysaccharides [48], and Sudan Red 7B for lipids [49].

#### 4.3.2. Electron Microscopy

For scanning electron microscopy, samples of the stem apex were dissected to expose young leaves and subsequently fixed under a slight vacuum in Karnovsky’s solution (pH 7.2 in 0.1M phosphate buffer; modified from Karnovsky [45]). The samples were then dehydrated in an increasing ethanol series, critical-point-dried using CO_2_ [50], mounted in aluminum stubs, and coated with a gold–palladium alloy for further visualization in a Quanta 200 (FEI Company, Eindhoven, The Netherlands) electron microscope. 

For TEM, just the apical portion of young leaves was sampled, assuming that the colleters observed in other positions were structurally and functionally similar. We sampled leaves bearing intact apexes and leaves where the distal portion started to show signs of senescence by turning brown. These samples were fixed in Karnovsky’s solution (pH 7.2 in 0.1M phosphate buffer; modified from Karnovsky [45]) for 24 h and post-fixed in 1% osmium tetroxide for two hours. The fixed material was then dehydrated in an acetone series and embedded in epoxy resin [51]. Ultrathin sections were obtained using an ultramicrotome (UC6, Leica, Wetzlar, Germany) and contrasted with uranyl acetate and lead citrate [52,53]. Sections were observed under a Tecnai G2–Spirit (Philips/FEI) electron microscope.

## 5. Conclusions

Our data showed, for the first time, the presence of colleters in *A. andraeanum*, and, in turn, revealed a novel feature for the Araceae. Nonetheless, such glands in *Anthurium* do not seem to be restricted to *A. andraeanum*. Based on their structure, aspect, and position, we believe the *Vorläuferspitze* described by Henriquez [32] might bear terminal colleters in at least three additional species. Our observations also suggest senescent apical structures within the precursor tip of several *Anthurium* species, which may constitute colleters similar to those observed in *A. andraeanum*. Also, the “axillary squamules” reported for some Araceae species need further attention. These observations might suggest a wide occurrence of colleters within *Anthurium* and other Araceae. Future research, mainly correlating secretory activity in colleters of species that occupy different habitats, seems necessary to reveal the diversity of mucilage glands and their function and evolutionary significance in the family. The chemical nature of the colleters exudates that we observed in *Anthurium* seems to reinforce the idea that colleters of monocots differ from those of eudicots in this aspect, so we suggest that attention be paid to the chemical characterization of exudates.

## Data Availability

The data presented in this study are available on request from the corresponding author.
